# Ending Tribalism in Global Oncology

**DOI:** 10.1200/JGO.18.00171

**Published:** 2018-10-22

**Authors:** Satish Gopal

**Affiliations:** **Satish Gopal**, University of North Carolina at Chapel Hill, Chapel Hill, NC; University of Malawi College of Medicine, Blantyre; and Malawi Cancer Consortium & Regional Center of Research Excellence for NCDs, Lilongwe, Malawi.

My recent experiences suggest there is broad consensus that global health is important and that this is a settled issue, despite seeming not so settled at times during the last year. Foremost among these experiences was the opportunity to participate in the Fogarty International Center 50th Anniversary Scientific Symposium on May 1, 2018, which showcased the remarkably broad political, academic, and civil society support enjoyed by the center. It also highlighted the amazing constellation of human capacity building, health systems strengthening, and scientific achievements it has engendered around the world in its first 50 years through small, strategic, catalytic investments. Within my own global oncology discipline, a similar broad consensus—namely, that global health matters—has been demonstrated by recent advisory group discussions at the US National Cancer Institute regarding its substantial global health portfolio, the convening by ASCO of an Academic Global Oncology Stakeholders Summit, and a dedicated session highlighting cancer as a global issue at the annual meeting of the Association of American Cancer Institutes. I have been fortunate enough to be invited to participate in all of these gatherings.

Presuming, then, that global health is good because it produces broadly applicable knowledge and/or breakthroughs, as well as helps to improve the lives of fellow human beings, a follow-up question is whether all global health is good (no), and whether global health can be done better (yes).

In 2018, it is important to acknowledge that there is a particular type of condescension that is still surprisingly prevalent in academic global health programs in low- and middle-income countries (LMICs) and the remarkably colonial feel that many such programs still have. American universities are perhaps most culpable—in sub-Saharan Africa, these institutions often leverage their ample resources and scientific expertise to extract highly self-serving agreements from local institutions and governments, which are desperate to address the public health needs of their citizens. To those of us living here, the region can often feel divided into clearly marked university territories and/or satellite campuses, with tense demilitarized zones between them. Local staff have usually learned to cautiously navigate these borders to avoid self-harm. Within and across boundaries, there is often intense and even ruthless competition for patients, staff, space, biospecimens, data, and investigators. Often these battles are choreographed from afar by individuals with limited global health credentials, who are more often than might be reasonably predicted senior, white, male scientists who have spent little time themselves living and working in LMICs.

Everyone claims to not want such a situation, yet this continues to regularly occur and therefore must have certain advantages. There may be an analogy in the resurgent bilateral zero-sum approach to foreign policy, which has national self-interest as its clearly stated main priority and which lends itself more readily to short-term wins that are more easily demonstrable within the time horizon of election cycles. Grant cycles and time horizons for academic productivity can be equally unforgiving, and a similarly aggressive bilateral approach to global health partnerships may be attractive to speed progress toward more transactional and less lofty goals.

Multilateral global health partnerships, on the other hand, are ponderous, inefficient, and risky. Progress requires constant negotiation, and credit for achievements must be shared in a manner that does not come naturally to many academics, who are ultimately judged by the number and impact of their PubMed citations. But the scientific community has long understood that once the machinery and relationships are in place, these partnerships can be transformative. This is why cooperative groups and research networks exist. This is why breaking down silos and data sharing are central pillars of accelerating advances through the Biden Cancer Initiative.^1^ This is why almost all children with cancer in high-income countries are now cured of their disease.^2^

In sub-Saharan Africa specifically, achieving meaningful multilateral partnerships is especially difficult, given the abundance and diversity of foreign partners and various languages, customs, resource levels, and regulations across national borders. A primary obstacle is severely limited in-country human and material resources, which places local investigators and institutions in weak negotiating positions to effectively dictate terms of collaboration to foreign partners. This situation has often led to highly diluted science (eg, multiple parallel sequencing initiatives of small numbers of unique tumor types from different countries), such that results are hypothesis-generating and rarely definitive. Much more tragically, this situation has led to few advances for patients with cancer. For example, during the last 50 years, there has been near-stagnation in treatment and outcomes for African children with Burkitt lymphoma, who have not enjoyed nearly the same access to harmonized multicenter treatment protocols as children in high-income countries.^3^

This situation can be difficult to overcome without incentives and/or coercion to change individual and institutional behavior. Funding agencies probably have an important role to play, by bringing diverse multidisciplinary groups together for true global health team science to accelerate progress and maximize return on their investments. Shirking this responsibility would be an immense opportunity squandered, particularly for new global health areas such as noncommunicable diseases, where collaborative networks are still nascent without the scale, robustness, or maturity of those already in place for HIV and other infectious diseases. Institutional leaders may also need to revisit decision making and organizational structures within their global health programs, which routinely over-prioritize and over-reflect the world views of senior American and European scientists. No matter how renowned these individuals are, their world views can unfortunately be narrow in many respects and must be appropriately balanced.

To conclude, whether we are fundamentally tribes or humankind—or what precise mixture we are of both—is an ongoing and difficult debate. However, just as in diplomacy or national security arenas, it is naïve not to recognize a powerful version of this debate lurking beneath the surface of much current global health activity, nor tribalism’s surprising strength within this debate, nor the important consequences of this debate for care and research around the world. The delusion that colonial approaches to global health are of a bygone era is attractive and comforting. However, LMIC investigators and institutions understand better than anyone that these attitudes are alive and well, and require constant resistance.
